# Can C-reactive protein predict coagulation in off pump coronary artery bypass grafting? A cohort study

**DOI:** 10.1186/s13019-022-01949-w

**Published:** 2022-09-02

**Authors:** Xiaojie Liu, Yang Yu, Lijuan Wang, Sudena Wang, Yuchen Gao, Hushan Ao

**Affiliations:** 1 Department of Anestheiology, the Affiliated Hospital of Qingdao University, No. 59, Haier Road, Qingdao, Shandong province China; 2 Department of Anesthesiology, Fuwai Hospital, National Center for Cardiovascular Diseases, Chinese Academy of Medical Sciences and Peking Union Medical College, No.167 North Lishi Road, Xicheng District, Beijing, 100037 China

**Keywords:** Coronary artery bypass grafting, C-reactive protein, Bleeding, Coagulation

## Abstract

**Background:**

Previous study found that C-reactive protein (CRP) can predict bleeding after on-pump CABG. To evaluate whether preoperative C-reactive protein (CRP) can be a novel marker of postoperative bleeding in patients having off-pump coronary artery bypass grafting (CABG).

**Methods:**

This is a retrospective cohort study. Multiple variable regression analyses were performed. 537 patients undergoing off-pump isolated primary CABG at Fuwai Hospital from September 2017 to July 2018 were recorded. The primary endpoint was bleeding volume within 24 h after surgery.

**Results:**

Data of 537 patients undergoing off-pump isolated primary CABG at Fuwai Hospital were recorded. The correlations between bleeding volume within 24 h after surgery and preoperative data were analyzed with univariate and multivariate linear regression. Much more preoperative CRP concentration (B = −0.089, *P* < 0.05) was associated with less postoperative bleeding volume and fibrinogen (B = 0.594, *p* < 0.001).

**Conclusions:**

Preoperative CRP concentration is independently correlated with the postoperative volume of bleeding within 24 h. CRP may become a novel coagulation index in coronary artery atherosclerotic disease.

## Introduction

Postoperative bleeding remains a focused issues in cardiac surgery. Previous studies illustrated that several factors contribute to excessive bleeding after cardiac surgery, including preoperative drugs (including anticoagulants and antiplatelet drugs), cardiopulmonary bypass (CPB), coagulation factors, hyperfibrinolysis, and residual heparin effects. The traditional indicators are hemoglobin, platelets, and basic anthropometric indicators [1–3], such as sex, age, body mass index (BMI), ejection fraction (EF) within 30 days, recent myocardial infarction, unstable angina,heart failure, active infective endocarditis, creatinine (Cr), and preoperativeuse of anticoagulation and antiplatelet drugs. Emergency surgery, reoperation, CABG and valve surgery and large blood vesselsare some of the surgical indicators [4].

A large multicenter clinical study illustrated that other new indictors of postoperative bleeding, such as preoperative thrombocytopenia [5].Preoperative fibrinogen concentration (even within the normal range) is a limiting factor for bleeding after CABG [6]. Previous retrospective study and other studies found that BMI is an important predictor of bleeding after CABG [7, 8].

CRP is a common inflammatory biomarker in clinical practice. As a risk factor for atherosclerosis, it is correlated with the risk of cardiovascular disease (CVD) events such as myocardial infarction (MI). Lorenzo’s et al. have illustrated that CRP can increase the risk of death 11.7 times after CABG [9]. A large number of clinical studies have shown that CRP > 3 mg/L is the threshold for increasing the risk of cardiovascular risk. Previous study illustrated that CRP participate in venous vascular bridge obstruction postoperatively after CABG. CRP can induce the expression of tissue factor and fibrinolytic enzyme activator inhibitor-1 and the promotion of fibrin in vein bridge vascular deposits as it can be synthesized systemically rather than locally. Animal studies have shown that CRP can increase the expression of tissue factor (TF) and decrease the expression of TF pathway inhibitors (TFPIs) [10]. TF, as the cell surface receptor of coagulation factor VIIa (FVIIa), initiates the coagulation cascade and forms a hemostasis capsule around important organs to participate in hemostasis [11]. The tissue factor-factor VIIa complex (TF/FVIIa) activates coagulation factor VIII (FVIII) and upregulates its activity.

Therefore, we speculated that CRP might be involved in the coagulation process during CABG. Previous study found that CRP was another indicator of bleeding after on-pump CABG [12]. Cardiopulmonary bypass (CPB) may induce an inflammatory response, which may be involved in the coagulation process. CRP and its relationship with fibrinogen in patients undergoing off-pump CABG need to be further studied. The outcome of this study was postoperative bleeding volume within 24 h. The second outcome was fibrinogen.

## Subjects and methods

### Study population

**Fig. 1:**
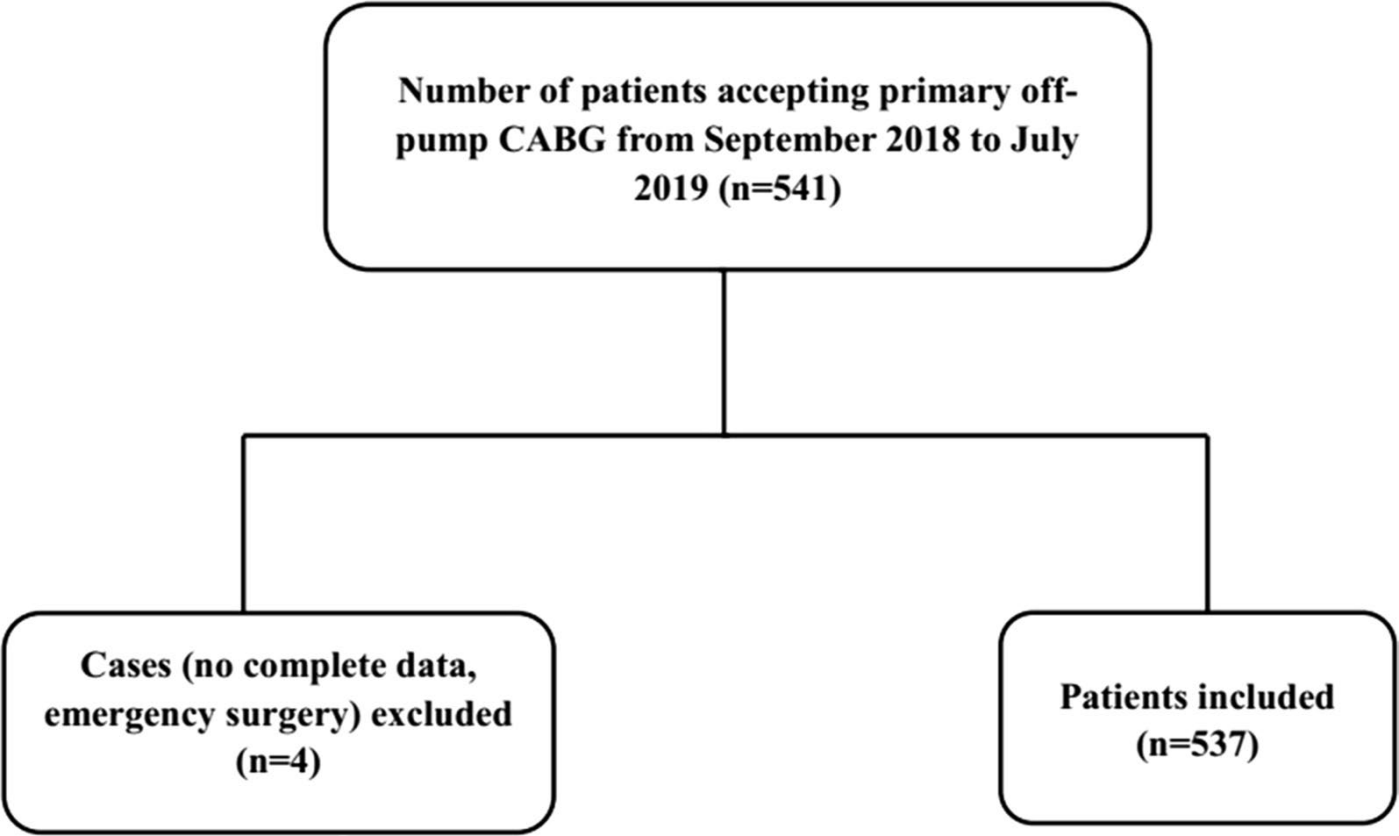
The flow chart of the patients included in this study

This was a retrospective study of consecutive patients who underwent isolated primary off-pump CABG at Fuwai Hospital. Isolated primary off-pump CABG was defined as the patient’s first coronary artery bypass graft surgery alone, without cardiopulmonary bypass. Data were collected from the Fuwai Hospital electronic medical records. The study was approved by the Ethics Committee of Fuwai Hospital, and written informed consent was waived. A total of 537 patients who consecutively underwent isolated, primary off-pump CABG from September 2018 to July 2019 at Fuwai Hospital in Beijing, China, were included in this study. The exclusion criteria of this study were as follows: patients with emergency CABG; on-pump CABG; concomitant surgical procedures; patients with acute infection status and liver and kidney dysfunction defined by the Goldman-Cecil Medicine [13]. After screening, 537 patients were selected, and 4 patients were excluded because of incomplete information (Fig. 1).

### Data collection

Patients’ basic characteristics, preoperative laboratory coagulation parameters [platelet (PLT) count, plateletcrit (PCT), prothrombin time (PT), activated partial thromboplastin time (APTT), D-dimer, fibrinogen and fibrinogen degradation product (FDP)], preoperative anticoagulant drugs, intraoperative hemostatic drugs, and postoperative bleeding volume within 24 h after surgery were recorded.

Postoperative bleeding volume was defined as the total amount of chest tube drainage within 24 h postoperatively. High CRP was defined as CRP > 3 mg/L, and normal CRP was CRP <  ≤ 33 [14].

### Statistical analysis

Categorical variables are expressed as numbers and percentages, and continuous variables are expressed as the mean ± standard deviation (for comparing normally distributed continuous variables between groups) and the median (IQR) (for comparing nonnormally distributed continuous variables between groups). To compare the characteristics between cohorts, we used the χ^2^ test for categorical variables and Student’s *t* test or Wilcoxon rank sum test for continuous variables, depending on the distribution.

Simple linear regression was used to analyze the relationship between demographic data and postoperative bleeding volume. A multiple linear regression model was used to identify the independent variables for postoperative bleeding volume. A covariate was included in the multiple linear regression models if its p value was less than 0.1 in univariate regression analysis.

All statistical analyses were performed with SPSS version 23.0 software (SPSS Inc., Chicago, IL, USA).

## Results

**Fig. 2:**
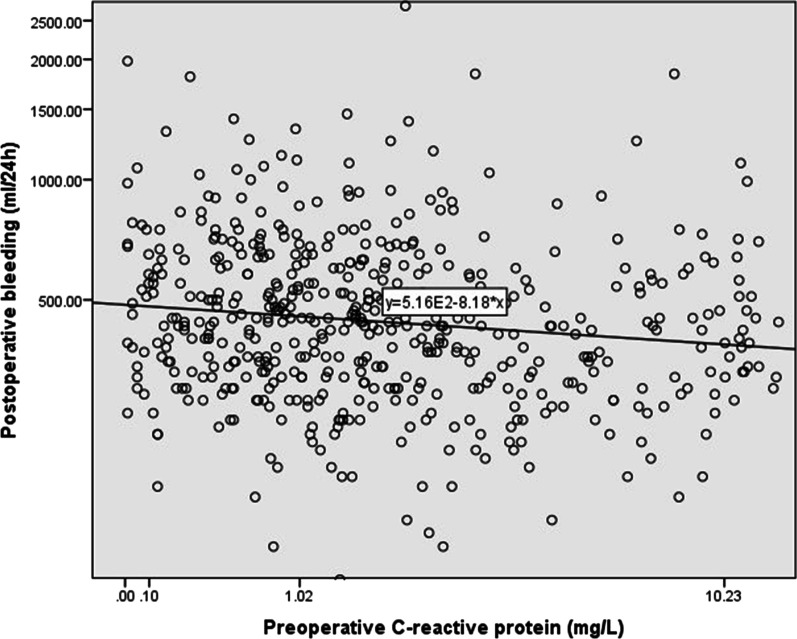
Correlation between C-reactive protein and postoperative bleeding

Table 1 presents the demographic and clinical data of the patients. Postoperative bleeding volume was significantly negatively correlated with preoperative CRP concentration (*p* < 0.05), tranexamic acid (*p* < 0.05), PLT count (p < 0.05), plateletcrit (PCT) (*p* < 0.001), age (*p* < 0.05), BMI (*p* < 0.05), HGB (*p* < 0.05) and fibrinogen (*p* < 0.05). Preoperative CRP concentration was an independent predictor of postoperative bleeding volume by multiple linear regression (*P* < 0.05) (Table 2, Fig. 2). After further multiple linear regression. FIB fibrinogen (*P* < 0.001) was positively correlated with preoperative CRP (Table 3).

**Table 1:** Baseline Characteristics of the Patients (n = 537)

Age (years)	60.6 ± 8.33
Male (%)	428 (79.7)
Hypertension (%)	337 (62.8)
Hyperlipidemia (%)	426 (81.6)
Diabetes (%)	203(37.8)
Smoking (%)	235 (54.9)
Arrhythmia (%)	19 (3.5)
LVEF (%)	60.20 ± 7.27
BMI (kg/m^2^)	25.94 ± 3.22
HGB (g/L)	134.59 ± 17.40
PLT count (× 10^9^/L)	214.96 ± 58.15
PCT (%)	0.23 ± 0.06
PT (sec)	13.21 ± 1.75
APTT (sec)	36.85 ± 7.35
FIB (g/L)	3.55 ± 0.83
D-dimer (μg/ml)	0.27 (0.21, 0.42)
FDP (μg/ml)	2.5 (2.5, 2.5)
Aspirin (%)	204 (3.8)
Ticagrelor (%)	24 (4.5)
Tranexamic acid (%)	354 (69.4)
CRP (mg/L)	1.52 (0.70, 3.11)
Postoperative bleeding (mL/24 h)	430 (320, 580)

*LVEF* left ventricular ejection fraction, *BMI* body mass index, *HGB* hemoglobin, *PLT* platelet, *PCT* plateletcrit, *PT* prothrombin time, *APTT* activated partial thromboplastin time, *FIB* fibrinogen, *FDP* fibrinogen degradation product, *CRP* C-reactive protein

**Table 2:** Linear regression analysis for postoperative bleeding volume of off-pump CABG

	Simple linear regression		Multiple linear regression	
	B	*P* Value	B	*P* Value
Age	0.098	0.023	0.106	0.030
Female (%)	0.076	0.079		
Hypertension (%)	−0.058	0.183		
Hyperlipidemia (%)	−0.002	0.955		
Diabetes (%)	−0.080	0.065		
Arrhythmia (%)	−0.021	0.629		
LVEF (%)	0.046	0.285		
BMI (kg/m^2^)	−0.148	0.001	−0.107	0.015
HGB (g/L)	−0.089	0.040		
PLT count (× 10^9^/L)	−0.110	0.011		
PCT (%)	−0.124	0.004		
PT	0.048	0.275		
APTT	−0.029	0.581		
FIB (g/L)	−0.086	0.117		
D-dimer (μg/ml)	−0.123	0.083		
FDP (μg/ml)	−0.121	0.162		
CRP (mg/L)	−0.088	0.044	−0.088	0.042
Aspirin (%)	−0.011	0.795		
Ticagrelor (%)	0.044	0.315		
Tranexamic acid (%)	−0.388	< 0.001	−0.381	< 0.001

*LVEF* left ventricular ejection fraction, *BMI* body mass index, *HGB* hemoglobin, *PLT* platelet, *PCT* plateletcrit, *PT* prothrombin time, *APTT* activated partial thromboplastin time, *FIB* fibrinogen, *FDP* fibrinogen degradation product, *CRP* C-reactive protein

**Table 3:** Linear regression analysis for preoperative FIB of patients

	Simple linear regression		Multiple linear regression	
	B	*P* Value	B	*P* Value
Age (years)	0.009	0.865		
Female (%)	−0.138	0.012	−0.139	0.003
BMI (kg/m2)	0.102	0.604		
HGB (g/L)	−0.105	0.057	−0.010	0.830
PLT count (× 10^9^/L)	0.094	0.086	0.043	0.322
PT(s)	−0.010	0.850		
APTT(s)	0.132	0.016	0.053	0.232
Smoking (%)	0.013	0.820		
CRP (mg/L)	0.604	< 0.001	0.594	< 0.001
Aspirin (%)	0.155	0.005	0.108	0.013
Ticagrelor (%)	0.031	0.577		
Tranexamic acid (%)	−0.070	0.240		

*BMI* body mass index, *HGB* hemoglobin, *PLT* platelet, *PCT* plateletcrit, *PT* prothrombin time, *APTT* activated partial thromboplastin time, *CRP* C-reactive protein

## Discussion

This study has shown that CRP was associated with postoperative bleeding within 24 h in patients undergoing off-pump CABG, separating from the influence of CPB and anticoagulant drugs.

CRP, a general marker of inflammation, mediates and predicts the development of vascular occlusive diseases such as myocardial infarction and stroke which thus predicts postoperative outcomes [15]. A previous study illustrated early postoperative C-reactive protein elevation and long-term postoperative major adverse cardiovascular and cerebral events in patients undergoing off-pump coronary artery bypass graft surgery [16]. Another study found that patients with high C-reactive protein were at significantly higher risk of mortality than those with low C-reactive protein without transfusion [17]. A meta-analysis illustrated that elevated baseline hs-CRP levels were independently associated with excessive ischemic stroke risk but exhibited no clear effect on hemorrhagic stroke [18].

Reports have described CRP was higher in patients with thrombotic complications than in those without. DIC, clinically relevant thrombocytopenia, and low fibrinogen are rare and have been associated with significant bleeding manifestations [19]. Further studies found that higher CRP increased fibrinogen and decreased the FVIII/VWF: Ag ratio at admission, which were significantly associated with the risk of increased oxygen requirement during follow-up [20]. The role of preoperative CRP as a biomarker of coagulation function in patients undergoing on-pump CABG has been reported by previous study. However, CPB induces coagulation system disorder more than other strategies, such as extracorporeal circulation prime. Thus, preoperative CRP concentration and coagulation parameters of patients undergoing off-pump CABG was studied rather than those of on-pump CABG to eliminate such interference.

In this study, patients accepting off-pump CABG were selected. This study further illustrated that preoperative CRP was negatively correlated with postoperative bleeding within 24 h. Previous studies have shown that hsCRP can promote monocyte-endothelial cell interactions and promote the formation of plasminogen activator inhibitor-1 (PAI-1) and tissue factor (TF), which act as cell surface receptors for coagulation factor FVIIA and initiate the coagulation cascade. A correlation between CRP and APTT was found in last study [21]. However, this study showed that there was no correlation between CRP and APTT, perhaps because the study sample was small.

A previous study illustrated that tranexamic acid reduced perioperative blood transfusion in cardiac surgery (Class 1A) [22]. This study illustrated that it can reduce postoperative bleeding, which was consistent with our previous study [23].

This study also demonstrated that preoperative CRP correlated with fibrinogen. As a reactive substrate, fibrinogen is converted into fibrin under the action of thrombin and becomes the main structural protein of blood clots. Like classic indicators, such as sex, age, BMI, EF, myocardial infarction within 30 days, unstable angina pectoris, heart failure, active infective endocarditis, preoperative Cr, HGB, use of anticoagulant and antiplatelet drugs, cardiac surgery type, and cardiopulmonary bypass [24], fibrinogen is associated with postoperative bleeding. Meta-analyses have shown that there is a significant correlation between preoperative fibrinogen levels and postoperative blood loss [25]^.^ Early administration of fibrinogen could reduce postoperative bleeding after complex pediatric cardiac surgery [26]. Fibrinogen has become another inflammatory marker following systemic inflammation markers, such as hsCRP, TNF-α and IL-6 [27]. An earlier study illustrated that assessment of the CRP or fibrinogen level in people at intermediate risk for a cardiovascular event participating in ischemic cerebrovascular events [28]. Elevated fibrinogen was independently associated with MACEs in CAD patients, especially among those with pre-DM and DM [29], which suggests that it may participate in metabolic syndrome with chronic low-grade inflammation. Studies have illustrated that CRP is associated with a 1.9% increase in γ′ fibrinogen after adjustment for potential confounders [30]. All of the above findings are consistent with our study.

Several limitations are worth noting. First, this study was conducted in a single center among ethnic Chinese patients, which may not reflect worldwide practice. Second, the surgical technique may affect the amount of postoperative bleeding. However, the surgical time and surgical procedure were consistent throughout our study.

In summary, this study illustrated less blood loss with elevated preoperative CRP concentrations in patients undergoing off-pump CABG. Preoperative CRP may be further used as a new coagulation indicator in addition to the standard laboratory coagulation index, which maybe can be used as the current supplement of bleeding scoring system.

## Data Availability

Data supporting the results reported in the article can be accessed by connecting aohushan@126.com.

## Abbreviations

APTT: Activated partial thromboplastin time
BMI: Body mass index
CRP: C-reactive protein
CABG: Coronary artery bypass grafting
CPB: Cardiopulmonary bypass
Cr: Creatinine
CVD: Cardiovascular disease
EF: Ejection fraction
FDP: Fibrinogen degradation product
MI: Myocardial infarction
TF: Tissue factor
TFPIs: Tissue factor pathway inhibitors
PLT: Platelet
PCT: Plateletcrit
PT: Prothrombin time
HGB: Hemoglobin
